# Therapeutic Applications of Ketogenic Diets in Lipedema: A Narrative Review of Current Evidence

**DOI:** 10.1007/s13679-025-00642-y

**Published:** 2025-05-26

**Authors:** Nevin Sanlier, Serra Baltacı

**Affiliations:** Department of Nutrition and Dietetics, School of Health Sciences, Ankara Medipol University, 06050 Altındag, Ankara Turkey

**Keywords:** Lipedema, Fat accumulation, Ketogenic diet, Ketosis, Inflammation

## Abstract

**Purpose of Review:**

Lipedema is an abnormal accumulation of adipose tissue, predominantly observed in women, characterised by symmetrical fat deposition and tactile sensitivity in the extremities, affecting both sides of the body. This condition can lead to significant pain, impairing daily activities and causing substantial discomfort.

**Recent Findings:**

While the etiology of the disease is not yet fully understood, genetic predisposition, hormonal fluctuations, a stressful lifestyle, as well as traumatic events are considered potential triggers. Lipedema remains a condition with low diagnostic awareness as well as is frequently misdiagnosed as obesity or lymphedema. While obesity is a risk factor for lipedema, the abnormal fat deposition characteristic of the disease can occur across a wide spectrum of body weights, from underweight to overweight individuals.

**Summary:**

Specific patterns of adipose tissue distribution may be associated with signs of inflammation as well as heightened pain perception, as well as individuals with eating disorders, such as anorexia, may additionally be affected. Ketogenic diets have emerged as a promising therapeutic option for lipedema. Characterized by low carbohydrate as well as high fat content, ketogenic diets facilitate metabolic improvements by reducing insulin resistance as well as supporting weight loss. Furthermore, they may mitigate tissue damage associated with lipedema by decreasing inflammation as well as oxidative stress levels.Nevertheless, current scientific data regarding the mechanisms of action as well as therapeutic efficacy of ketogenic diets are limited, necessitating further research to expand their clinical application.

## Introduction

Lipedema is a chronic disorder of adipose tissue of unknown etiology, primarily affecting women. It is distinguished from lymphedema by its characteristic features, including symmetrical fat accumulation in the lower extremities, pain, easy bruising with minimal trauma, the presence of a cuff sign at the ankles, hypersensitivity, the development of hard nodules in subcutaneous adipose tissue, and marked resistance to exercise and conventional diet therapy. A genetic predisposition is thought to contribute to its development, and lipedema is often misdiagnosed as obesity or lymphedema. While obesity can exacerbate lipedema, it remains a distinct clinical entity [1–3]. The etiology and pathophysiology of lipedema are not fully understood. Factors such as hormonal changes during puberty, pregnancy, and menopause, stressful lifestyle alterations, surgical interventions, or trauma are believed to trigger the condition [4–6]. Patients with lipedema commonly experience bruising, heaviness in the legs, and heightened tactile sensitivity [7]. If left untreated, lipedema may lead to obesity, reduced mobility, decreased quality of life, and psychological dysfunction [8, 9]. Nevertheless, according to the latest German S2 guideline on lipedema (2024), there is no evidence that lipedema causes obesity or vice versa. The guideline clearly states that lipedema is neither caused by obesity nor does it cause obesity. However, it is noted that obesity can exacerbate the severity of lipedema [10]. A low-carbohydrate, high-fat ketogenic diet appears to be an effective approach for promoting weight and fat mass reduction in women with lipedema. Observed metabolic improvements include reductions in baseline insulin levels, decreases in the HOMA-IR, and diminished levels of inflammation and oxidative stress [11]. Such dietary interventions have been associated with significant decreases in body weight, BMI, and pain sensitivity [12]. A systematic review reported that women following KD protocols for approximately 15.85 weeks exhibited a mean BMI reduction of 4.23 kg/m2, an average weight loss of 7.94 kg, and decreased waist and hip circumferences, alongside improvements in body composition [13]. In a controlled study, a low-carbohydrate diet significantly reduced calf subcutaneous adipose tissue area and pain, highlighting its potential therapeutic benefits [14]. The anti-inflammatory effects of ketogenic diets are also considered beneficial in alleviating symptoms of lipedema [15]. Further studies have reported improvements in pain reduction, body weight loss, and quality of life following ketogenic interventions [14]. Personalized approaches, such as combining KD with carboxytherapy, have shown even greater efficacy in improving body composition and reducing pain [16]. Although ketogenic diets represent a promising approach in the management of lipedema, it is important to note that individual responses can vary, and more longitudinal studies are required to assess their long-term efficacy and safety. In addition to diet, comprehensive treatment approaches including pharmacological, surgical, psychological, and physical therapy interventions remain critical for effective disease management.

This review aims to investigate the potential mechanisms of action, beneficial properties, and clinical implications of KD in lipedema. By synthesizing findings from in vitro, animal, and human studies, the review critically examines both the advantages and limitations of ketogenic dietary therapy. Additionally, a daily ketogenic meal plan is presented based on a case study, with calculated energy and nutrient values tailored for lipedema management.

## Methodology

In the pursuit of ameliorating lipedema and its associated symptoms, the ramifications of specific nutritional strategies, particularly those employing ketogenic dietary methodologies, on the quality of life and recuperation of affected individuals are garnering significant scholarly interest. Within this framework, a thorough literature review was executed to evaluate the influence of ketogenic diets on the therapeutic management of lipedema. The literature search was conducted utilizing the PubMed/Medline, PsycINFO, Web of Science, Scopus, ScienceDirect, and Google Scholar bibliographic databases. Investigations published in the English language from 2018 to 2025 were included in the assessment. The subsequent keywords were employed throughout the search process:"lipedema,""fat accumulation,""ketogenic diet,""ketosis,""obesity,""inflammation,""pain,""edema,""bruising,""hormone,""insulin,""fat metabolism,""leg,""cellulite,"and"painful cellulite."Authors performed searches and full texts were reviewed according to the following inclusion criteria: (1) publications dated from 2018 to 2025 (2) availability in full-text format in English, (3) classification as original research articles, reviews, systematic reviews, meta-analyses, letters to the editor, clinical human studies, animal studies, in vivo*, *in vitro research, cell-based animal and human studies.

Studies published in non-English languages ​​or those in preprint stages were excluded from the review.A total of 93 references were analyzed based on the specified inclusion criteria, and the findings from seven key studies were summarized in a tabular format. The studies were accessed using the databases and search strategies described above.

## Etiology and Epidemiology of Lipedema

Lipedema is characterized by the abnormal proliferation of adipose tissue, primarily triggered by hormonal changes, leading to fat accumulation predominantly in the lower body [7, 17]. It is estimated that approximately 10% of women are affected by lipedema, although it is rarely observed in men [18]. Globally, lipedema is believed to impact approximately one in nine adult women [19]. Common clinical features include easy bruising, pain, impaired mobility, and hypersensitivity to touch, all of which contribute to significant functional limitations and reduced quality of life [20]. In approximately 80% of cases, the upper extremities are also affected, although the hands and feet typically remain uninvolved [1]. Pain is considered the primary symptom and is associated with allodynia, heightened sympathetic nerve signaling, and estrogen-mediated mechanisms. Although the precise mechanisms underlying pain are not fully elucidated, effective treatment can provide significant symptom relief.

The most minimally invasive and currently effective treatment for lipedema is microcannulated tumescent liposuction, which has been shown to substantially reduce pain in affected patients [21]. While weight gain can exacerbate the symptoms of lipedema, there is no evidence suggesting that weight gain alone causes the disease. Rather, weight gain may serve as a trigger in genetically predisposed individuals [22, 23]. Traditional weight loss strategies, including intense physical activity and caloric restriction, are often ineffective at reducing lipedema-associated fat, sometimes exacerbating the disproportion between the upper and lower body [7]. Diagnosis of lipedema remains challenging due to the heterogeneity of its clinical presentation and the lack of objective diagnostic tools [24]. Notably, significant differences have been documented in leg fat mass between women with lipedema and obese women without the condition [25].

Revised diagnostic criteria for lipedema emphasize features such as gynoid fat deposition resistant to weight loss, bilateral lower limb swelling that persists despite elevation, and the presence of palpable nodules under the skin [4]. Lipedema is classified into three progressive stages: Stage I: Small nodules and reversible edema, Stage II: Larger, nut-sized nodules with reversible or irreversible edema, Stage III: Severe fatty deposits, macro-nodular changes, associated lymphedema, and a possible positive Stemmer sign [24, 26].

Differentiating lipedema from non-lipedema obesity is crucial. In typical obesity, fat is distributed in an android pattern, responds to conventional weight loss methods, and does not exhibit pain or edema [27]. Early and accurate diagnosis is vital, as misinterpreting lipedema as obesity may lead patients to harmful restrictive dieting practices, resulting in energy or protein-energy malnutrition [28].

**Fig. 1:**
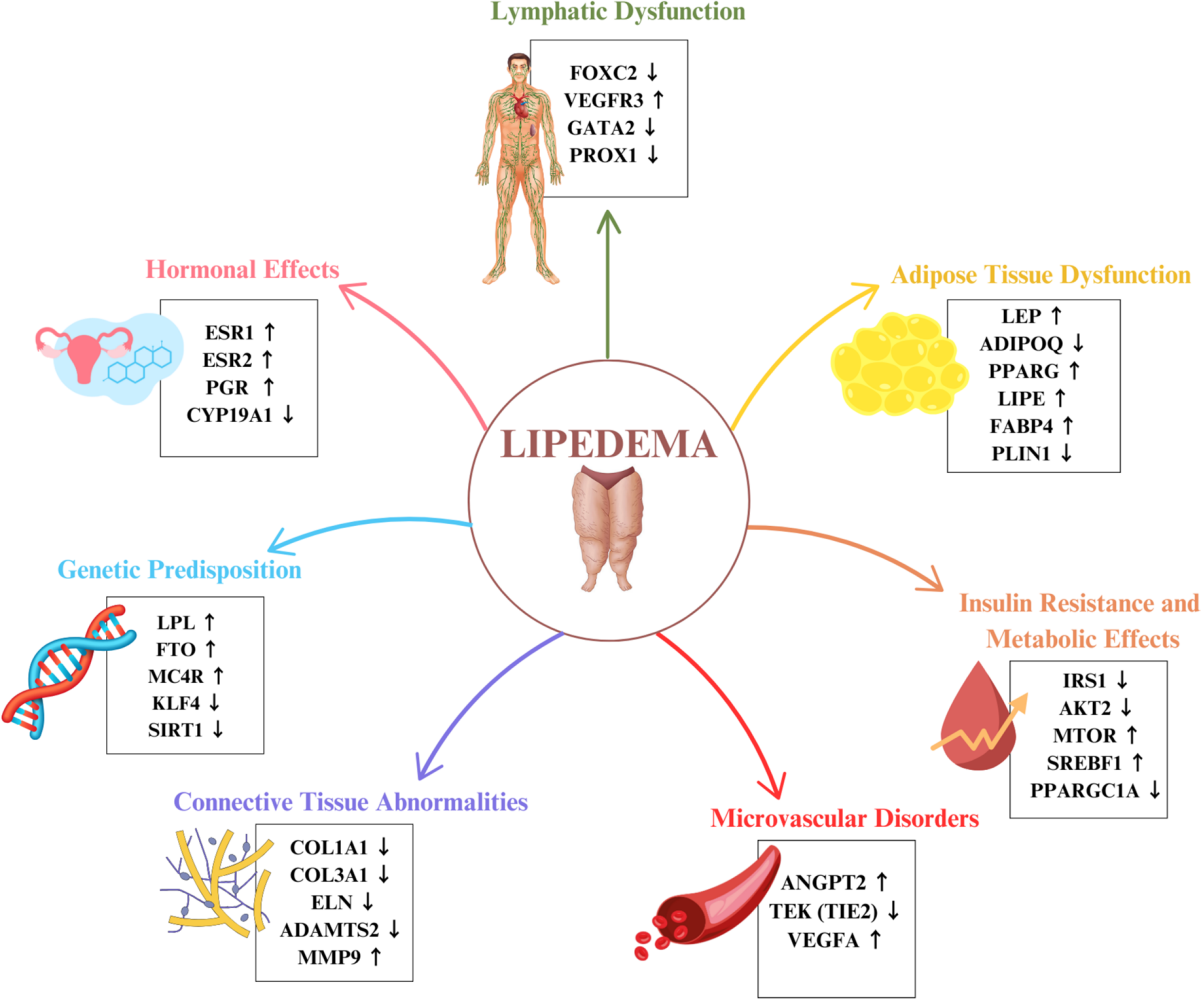
Various mechanisms and pathways and genes that increase and decrease lipedema

Various mechanisms and pathways and genes that increase and decrease lipedema are presented in Fig. 1.

## Pathophysiology of Lipedema

Despite the recent publication of extensive histologic and molecular genetic studies, the basic etiology and pathology of lipedema remain largely unclear [29]. Several hypotheses have been proposed regarding its pathophysiology, and it is thought that gene mutations associated with three main biological pathways may play a role in its somatic development. These are genes of leukocyte clones, genes related to mitochondrial activity, and genes related to localized disorders of subcutaneous adipose tissue [30]. It is also thought that steroid hormones may play a role in the pathogenesis of lipedema. Indeed, AKR1 C1, a gene encoding a protein involved in steroid hormone metabolism, was identified as the first suggestion to be associated with lipedema. The study by Kaftalli et al. provides evidence that AKR1 C1 may be a key gene in the pathogenesis of lipedema and that common polymorphisms may predispose to the development of lipedema [31, 32].

It is emphasized that lipedema should be considered as an endocrinological pathology. In addition to genetic predisposition, lipedema has been reported to be associated with significant hormonal changes, particularly abnormal expression of ERs in adipose tissue. In particular, downregulation of ER-α and upregulation of ER-β in affected areas have been identified as characteristic features [11]. Despite the proposed autosomal dominant mode of inheritance based on family tree analyses, it has been proposed that this disorder is caused by a polygenic-mediated change in the alpha and ER distribution pattern in white adipose tissue in the affected areas (ER-α expression ↓, ER-ß expression ↑) [24]. Another hypothesis suggests that lipedema is associated with macrophage accumulation and changes in macrophage polarization. A well-known protein superfamily contributing to macrophage polarization and accumulation is the MIF family. MIF-1 and MIF-2 regulate inflammatory processes in adipose tissue. In another study, mRNA expressions of MIF-1 and CD74 were significantly increased in patients with lipedema, while MIF-2 expression was unaffected, suggesting a possible contribution of the MIF family via the MIF-1-CD74 axis in lipedema [33]. Given that different populations of macrophages influence adipose tissue differentiation and metabolic processes, the use of the selective PI3 Kγ antagonist IPI-549 to induce macrophage polarization from M2 to M1 in lipedema, followed by the differentiation of adipose tissue-derived stem cells from IPI-549-treated SVF in conditioned medium, resulted in a significant reduction in lipid accumulation compared to control SVF in lipedema. The results suggest that CD163 + macrophages are a crucial factor in lipedema and that repolarization of lipedema macrophages may normally differentiate adipose tissue-derived stem cells in vitro when assessed by cell lipid accumulation [34]. The GH/IGF-1 pathway is known to have significant effects on adipocyte metabolism due to GH being a potent stimulator of lipolysis. However, these effects do not occur via IGF-1; IGF-1 plays a critical regulatory role in the terminal differentiation processes of adipocytes. There are no studies in the literature specifically examining the GH/IGF-1 pathway in patients with lipedema. However, in an in vitro study on fat stem cells, it was found that IGF-1 expression was significantly increased during the proliferation process in stem cell cultures obtained from patients with lipedema compared to the control group [35].

Another hypothesis regarding the pathophysiology of lipedema is the presence of primarily microvascular dysfunction in the blood capillaries and the lymphatic system. This is thought to lead to endothelial dysfunction and thus increased angiogenesis due to hypoxic stimuli resulting from excessive expansion of adipose tissue. An alternative view suggests that this disorder may result from a mechanical deficiency in lymph drainage. Increased capillary permeability causes proteins to escape into the extracellular compartment ("capillary leakage"), resulting in tissue edema [24]. According to another hypothesis, one of the main triggers of lipedema is the accumulation of bacterial lipopolysaccharides (endotoxins) in lower extremity fat deposits (gluteofemoral white adipose tissue; gfWAT) [36]. In addition, it has been suggested that interstitial fluid accumulation due to microvascular dysfunction may also play a role in the pathogenesis of lipedema. Disruption of interstitial fluid and tissue volume homeostasis is associated with increased interstitial fluid, tissue sodium, and adipose tissue accumulation in lipedema [27, 37]. Further studies have shown that edema, bruising, joint hypermobility, spider veins, cold-like symptoms, and fatigue are more commonly observed in individuals with lipedema [38]. These findings support the theory that lipedema is not merely a fat storage disorder but rather a tissue disorder associated with microvascular and neurological dysfunction [4]. Mast cells, immune cells that secrete histamine and other inflammatory molecules, mediate hypersensitivity and allergic reactions in the body. Based on the hypothesis that mast cells play a role in lipedema pathology, biopsies were taken from SAT in both lipedema and control groups and histologically analyzed for the presence of mast cells. Histological examination confirmed the presence of mast cells in lipedema tissue. Furthermore, metabolomic analysis revealed elevated histamine levels and its metabolites in lipedema samples compared to controls. After a two-week treatment, histamine levels in lipedema tissue were significantly reduced, suggesting decreased mast cell activity [30].

Moreover, it has been suggested that progesterone causes a slower and less efficient decrease in 20-α-hydroxyprogesterone, which may lead to increased subcutaneous fat accumulation. This opens up the potential for targeted pharmacological therapies for lipedema. Thus, understanding the underlying genetic factors in lipedema could be crucial for developing pharmacological treatments that directly affect the relevant or causal molecules [39].

## Mechanisms of Formation of Lipedema and Pathways

### Mechanisms of Lipedema Formation

*Hormonal Effects*; Lipedema is strongly associated with periods of hormonal fluctuation in women, notably during puberty, pregnancy, and menopause, thereby suggesting a critical role for estrogen in its pathogenesis. Estrogen is known to modulate adipose tissue metabolism and distribution, potentially through altered estrogen receptor expression and enhanced paracrine estrogen release from adipocytes. Dysregulation of estrogen signaling pathways may promote adipogenesis and lipid accumulation within adipocytes, contributing to the distinctive adipose tissue expansion observed in lipedema [26].

*Genetic and Molecular Factors*; A genetic predisposition to lipedema has been proposed, although specific genetic markers have yet to be conclusively identified. Altered gene expression patterns associated with lipid metabolism and cellular proliferation have been documented in lipedema tissues. Disruptions within key signaling networks, particularly the Bub1 signaling pathway, have been implicated in the hyperproliferation of adipose-derived stem cells, suggesting a molecular mechanism underlying increased adipogenesis in lipedema [40].

*Metabolic and Inflammatory Mechanisms*; Different metabolic profiles have been identified in lipedema patients, including altered levels of metabolites such as pyruvic acid, histidine, and phenylalanine, which may serve as potential biomarkers of the disease [41]. Inflammatory processes are also involved, with increased macrophage accumulation and altered macrophage polarization observed in lipedema tissues. The MIF family, particularly the MIF-1-CD74 axis, is involved in these inflammatory changes [33].

*Extracellular Matrix and Lymphatic Dysfunction;* Lipedema is associated with extracellular matrix remodeling and lymphatic dysfunction and contributes to characteristic swelling and pain. Dysregulation of proteins such as CAV1 and matrix metalloproteinase MMP14 can disrupt feedback mechanisms, leading to fat hypertrophy and vascular dysfunction [42]. In lipedema, lymphatic changes are noted, such as increased vascular area and regional lymphostasis, which can exacerbate fluid retention and inflammation [18]. Ongoing research into the hormonal, genetic, and metabolic basis of lipedema is important to develop targeted therapies and improve patient outcomes.

### Pathways of Lipedema Formation

*Platelet Transcriptome and Thrombosis*; Emerging evidence indicates that lipedema is associated with a prothrombotic phenotype, potentially linked to alterations in the platelet transcriptome. Patients with lipedema demonstrate distinct biological pathway profiles related to protein synthesis, degradation, and metabolism compared to individuals with lymphedema or obesity. These transcriptomic differences may underlie the elevated risk of venous thromboembolism observed in lipedema and underscore the necessity for further exploration of platelet activation mechanisms [43].

*Immune Cell Infiltration and Adipocyte Differentiation;* An increased infiltration of M2-polarized macrophages has been observed in lipedema tissues, significantly impacting adipocyte differentiation. Elevated numbers of CD163 + macrophages, involved in cytokine-mediated signaling and extracellular matrix organization, have been identified. Notably, pharmacological polarization of macrophages from the M2 to the M1 phenotype via PI3 Kγ inhibition has shown promise in restoring normal adipose-derived stem cell differentiation, presenting a potential therapeutic target [34].

*MicroRNA and Gene Expression;* Differential expression of specific microRNAs (miRNAs) in lipedema tissue has been implicated in the regulation of pathways involved in cell cycle control, insulin resistance, and inflammation. Upregulation of hsa-let-7 g-5p and downregulation of hsa-miR-205-5p have been documented, affecting the expression of target genes critical for cellular homeostasis [44]. These findings provide novel insights into the molecular underpinnings of lipedema.

*Adipocyte Heterogeneity and Signaling Network;* Single-cell RNA sequencing has revealed three distinct adipocyte populations in lipedema, each with unique gene signatures. These include lipid-producing, disease-catalyzing, and lipedemic adipocytes [45]. Dysregulated signaling networks, such as those involving the cell cycle regulator Bub1, drive the increased proliferation of adipose-derived stem cells in lipedema. Targeting these pathways may offer new therapeutic strategies [40].

*Hormonal and Metabolic Factors:* Estrogen plays an important role in the pathogenesis of lipedema and affects fat distribution and inflammation through its receptors. Hormonal changes during puberty, pregnancy, or menopause can trigger or worsen the condition [26]. Despite better regulation of glucose metabolism, lipedema patients have higher levels of cholesterol and inflammatory markers, indicating a complex interplay between metabolic and inflammatory pathways [46].

Lipedema is a chronic condition characterized by the abnormal accumulation of subcutaneous adipose tissue, primarily affecting women. Despite its frequent misdiagnosis as obesity, emerging research highlights distinct metabolic profiles in individuals with lipedema. Studies indicate that women with lipedema often exhibit better lipid profiles and reduced markers of insulin resistance compared to those with obesity, even when matched for BMI. This suggests that lipedema may not carry the same metabolic risks typically associated with obesity, such as type 2 diabetes and metabolic syndrome.

Research indicates that women with lipedema have better glucose metabolism regulation, as evidenced by lower HbA1c levels compared to BMI-matched controls [46]. Additionally, studies have shown that lipedema patients often have lower levels of total cholesterol, triglycerides, and glucose, despite having a high BMI [41]. Although lipedema is associated with increased levels of circulating inflammatory and oxidative stress markers, these do not seem to impair glucose metabolism, suggesting a unique metabolic adaptation in lipedema [46]. Lipedema is characterized by a distinct cytokine profile, with increased levels of certain interleukins, which may influence metabolic activity and contribute to the condition's unique metabolic phenotype [47]. Lipedema is often misdiagnosed as obesity or lymphedema due to overlapping symptoms, such as disproportionate fat distribution and swelling [31]. The lack of specific biomarkers further complicates accurate diagnosis [5, 47].

While the understanding of lipedema pathways is advancing, challenges remain in distinguishing it from similar conditions such as obesity and lymphedema. The lack of specific biomarkers makes it difficult to diagnose, often relying on clinical assessment [24]. Further research into molecular mechanisms and potential therapeutic targets, such as those identified in recent studies, is important to improve patient outcomes.

### Lipedema Comorbidities

Lipedema may be complicated by lymphedema later in life. It is classified as primary or secondary. Primary lymphedema is caused by intrinsic abnormalities such as hypoplasia or dysfunction of the lymph vessels, while secondary lymphedema usually develops due to extrinsic factors such as surgical lymphadenectomy. It is characterized by bilateral and asymmetric swelling of both lower extremities with interstitial fluid accumulation due to inadequate drainage of fluid, cells, and proteins."Stemmer's sign,"a pathognomonic sign for lymphedema, is defined by the inability to lift the skin fold at the base of the second toe [48]. Additionally, the identification of platelet factor-4 as a potentially useful biological marker for lymphedema and lipedema suggests that there may be overlapping mechanisms underlying the pathophysiology of these two conditions [49].

Given that poorly managed lipedema can progress to the development of lipo-lymphedema, proper disease management is of paramount importance. This management includes two main objectives: first, to relieve subjective discomfort such as pain; and second, to prevent complications such as lipo-lymphedema, skin infections, psychological morbidities, gait disturbances, and joint deformities [50]. Common comorbidities of lipedema include cardiovascular diseases such as high blood pressure, disorders of the thyroid gland such as hypothyroidism, fibromyalgia, and type 2 DM [51]. In addition, Seefeldt et al. reported that menstrual irregularities, insomnia, and migraine are common in patients with lipedema [52]. In another study, disproportionate accumulation of lipedema tissue in the inner leg was shown to increase the risk of meniscal damage and osteoarthritis by causing valgus stresses, restricting knee flexion, and increasing the risk of falls by altering gait mechanics [53]. Considering the edema, gait changes, and symptoms of chronic venous disease associated with obesity, it is difficult to determine precisely to what extent lipedema and obesity contribute to clinical manifestations [38]. Lipedema cannot be effectively treated with bariatric surgery. In one study, no significant improvement was achieved in the characteristic pain symptoms of lipedema, despite patients losing an average of more than 50 kg [54]. Increased pain perception in lipedema has been associated with irregularities in local sensory nerve fibers through inflammatory processes. This cannot be explained by the mechanical compression of nerve fibers by expanding adipose tissue, as pain is not observed in other types of lipohypertrophy or lymphedema [24].

It has been reported that there is a significant relationship between the development of obesity and the emergence of lymphedema in individuals with lipedema [54]. It has been reported that the most common comorbidity in lipedema is obesity. In a study conducted on Swedish women diagnosed with lipedema, it was found that individuals were overweight and obese [55]. Women with lipedema are at high risk of becoming morbidly obese. Obesity itself is a risk factor for lymphedema and can trigger lipedema and accelerate its progression [1, 23]. Nemes et al. found that left atrial and left ventricular dimensions were larger, as well as ejection fraction, in patients with lipedema [56]. Melander et al. found that the persistent feelings of weight and pain in the legs experienced by women with lipedema lead to feelings of weakness and loss of control over the body [57]. Another study found that it is difficult to reduce excess fat in patients with lipedema using traditional weight loss techniques such as lifestyle changes, bariatric surgery, and pharmacological interventions [58]. The development of depression, appearance-related distress, self-hatred, low quality of life, and social isolation are quite common in patients with lipedema [48]. It is also reported that patients with lipedema have psychological difficulties, can become depressed, and are stressed by their appearance [8, 51, 59].

As general nutritional recommendations in lipedema, short-term diets should be strictly avoided, as they can often cause the yo-yo effect. The balance of energy intake and expenditure should be considered. Patients should be educated about adequate, balanced, and healthy nutrition and food diversity that they can follow sustainably for the rest of their lives. To reduce hyperinsulinemia, there should be no more than 4–6 h between meals, and attention should be paid to eating little and often. Sugar and sugar-containing foods and snacks and processed foods that increase blood glucose levels should be avoided [23, 60]. Consumption of healthy fats should be increased, trans fats should be avoided, and physical activity should be increased. Body weight should be monitored, controlled, managed, and tracked [1, 23]. In recent years, many dietary models and patterns have attracted attention in the medical nutrition treatment of lipedema. One of them is the ketogenic diet.

### Characteristics of Ketogenic Diets

KD is defined as an extremely low-carb, high-fat, and adequate or low-protein diet that promotes the production of ketone bodies [61]. Glucose, initially stored in the form of glycogen, can be used as an energy source. However, after three to four days, this storage is depleted, and fats in the body can be broken down into free fatty acids, providing raw material for ketone production in the liver [62]. The adult brain uses glucose as its main energy source, which accounts for about 2% of body weight and meets about 20–23% of the body's total energy needs [63]. KD increases the production of ketone bodies in the body, leading to a state of nutritional ketosis. This causes the body to use ketone bodies instead of glucose as the main energy source for vital processes [64]. When carbohydrate intake is insufficient, the oxidation of fatty acids peaks in mitochondria in hepatocytes, and acetyl-CoA production increases. Acetyl-CoA is then combined with oxaloacetate and enters the citric acid cycle. When oxaloacetate is depleted and its amount does not reach equilibrium in the citric cycle, acetyl-CoA is converted to ketone bodies as an alternative energy source for tissues outside the liver. These ketone bodies are produced by the liver in two main forms, acetoacetate and βHB [65]. Acetoacetyl-CoA combines with the enzyme HMG-CoA synthase to form HMG-CoA. HMG-CoA is cleaved to acetyl-CoA and AcAc by the enzyme HMG-CoA lyase. Acetoacetate is reduced to BHB by the action of BHB dehydrogenase or converted to acetone and excreted in breath and urine [66]. These ketone bodies are known for their ability to control substrate utilization, inflammation, oxidative stress, catabolic processes, and gene expression [67]. Ketone bodies produce more ATP than an equivalent amount of glucose [68].

Current ketogenic diet treatment modalities fall into four main groups: classical ketogenic diet, modified Atkins diet, medium-chain triglyceride ketogenic diet, and low glycemic index therapy [69]. KDs are given in ratios such as 3:1, 2:1, or 1:1 and are called modified KDs, determined according to the patient's age, individual tolerance, target ketosis level, and protein requirements while preparing the nutrition plan [70]. The difference between these diets is that the type and amount of fat they contain are different, and it has been reported that there is no significant difference in terms of effectiveness between these diet types [71]. Many potential mechanisms reveal the anti-inflammatory potential of the ketogenic diet. Firstly, the KD puts the body into a state of dietary ketosis. The processes that occur during ketosis have a systemic anti-inflammatory effect that directly impacts CVD. The second most significant factor is the removal of pro-inflammatory sugars from the diet. This directly impacts CVD. Restricting the total amount of carbohydrates in the diet can provide specific anti-inflammatory benefits in the cardiovascular metabolic health context. A high-fat, well-composed ketogenic diet is also rich in omega-3 fatty acids, whose anti-inflammatory and cardioprotective effects are well known [72]. A traditional KD is a 4:1 formulation of carbohydrate plus protein to fat content. A classic 4:1 KD provides 90% of calories from fat, 8% from protein, and only 2% from carbohydrates [73]. To improve flexibility and palatability, less strict KD variants have been developed in recent years, including lower ratio KDs, modified Atkins diet (MAD), low glycemic index therapy (LGIT), and combining these diets with medium-chain triglyceride (MCT) fat [70]. Ketogenic diets are a type of diet rich in fat and low in carbohydrates and protein, and the contents of ketogenic diets in clinical use are given in Table 1. A comparison of 4 basic ketogenic diets used clinically (1000 kcal/day) is given in Table 2.

**Table 1:** Energy amounts per dietary unit [92]

Ketogenic diet rate (fat/protein + carbohydrate)	Energy from fat (kcal)	Energy from carbohydrates and protein (kcal)
1.0:1	1 g × 9 = 9	1 g × 4 = 4
1.5:1	1.5 g × 9 = 13.5	1 g × 4 = 4
2.0:1	2 g × 9 = 18	1 g × 4 = 4
2.5:1	2.5 g × 9 = 22.5	1 g × 4 = 4
3.0:1	3 g × 9 = 27	1 g × 4 = 4
3.5:1	3.5 g × 9 = 31.5	1 g × 4 = 4
4.0:1	4 g × 9 = 36	1 g × 4 = 4

**Table 2:** Comparison of four basic ketogenic diets in clinical Use (1000 kcal/day)

Diet	Fat (g)	Protein (g)	Carbohydrate (g)
MCT fat diet	78	25	50
Low glycemic index diet	67	40–60	40–60
Modified Atkins diet	72	68–78	10–20
Classic long chain TG			
4:1	100	17	8
3:1	96	18	14
2:1	92	20	26
1:1	77	37	40

### Mechanisms of Ketogenic Diets

Given the progressive characteristics of diseases such as lipedema. early diagnosis and treatment are of great importance. These diseases can lead to a significant decrease in quality of life and immobility [74]. Conventional weight loss treatments are often inadequate in patients with lipoedema due to inflammation and fibrosis of the affected adipose tissue [75]. Ketogenic diets induce a state of metabolic ketosis in which the body shifts from relying on glucose to using ketone bodies as the primary energy source. This shift is associated with increased fat oxidation and decreased fat storage, which may contribute to the reduction of subcutaneous adipose tissue in lipedema [15, 76]. A low-carbohydrate diet has been shown to specifically target SAT in the lower limbs, a key feature of lipedema. A randomized controlled trial found that a low-carbohydrate diet reduced calf SAT area and circumference compared to a low-fat diet [14].

Lipedema a is associated with chronic inflammation, which contributes to disease progression and symptom severity. Ketogenic diets have been shown to reduce systemic inflammation by lowering levels of proinflammatory cytokines such as TNF-α and IL-6 [15, 76]. A high-fat ketogenic diet was reported to be more effective in reducing anthropometric measurements and improving symptoms in lipedema patients [77]. Ketogenic diets may also contribute to increased pain sensitivity and reduced swelling in affected areas [2, 76, 78].

It has been suggested that very low-calorie ketogenic diets, particularly in the context of obesity, may be more effective than other dietary approaches such as the Mediterranean diet or intermittent fasting in the treatment of lipedema [79]. Ketogenic diets have found significant improvements in short-term body weight, glucose tolerance, liver function, and lipid profiles in patients with lipedema and no adverse effects on kidney or thyroid function [80]. In addition, it is known to improve insulin sensitivity by reducing carbohydrate intake. This leads to lower insulin levels and improved fat metabolism. Improved insulin sensitivity may play a role in reducing the abnormal fat accumulation seen in lipedema [44, 81].

**Fig. 2:**
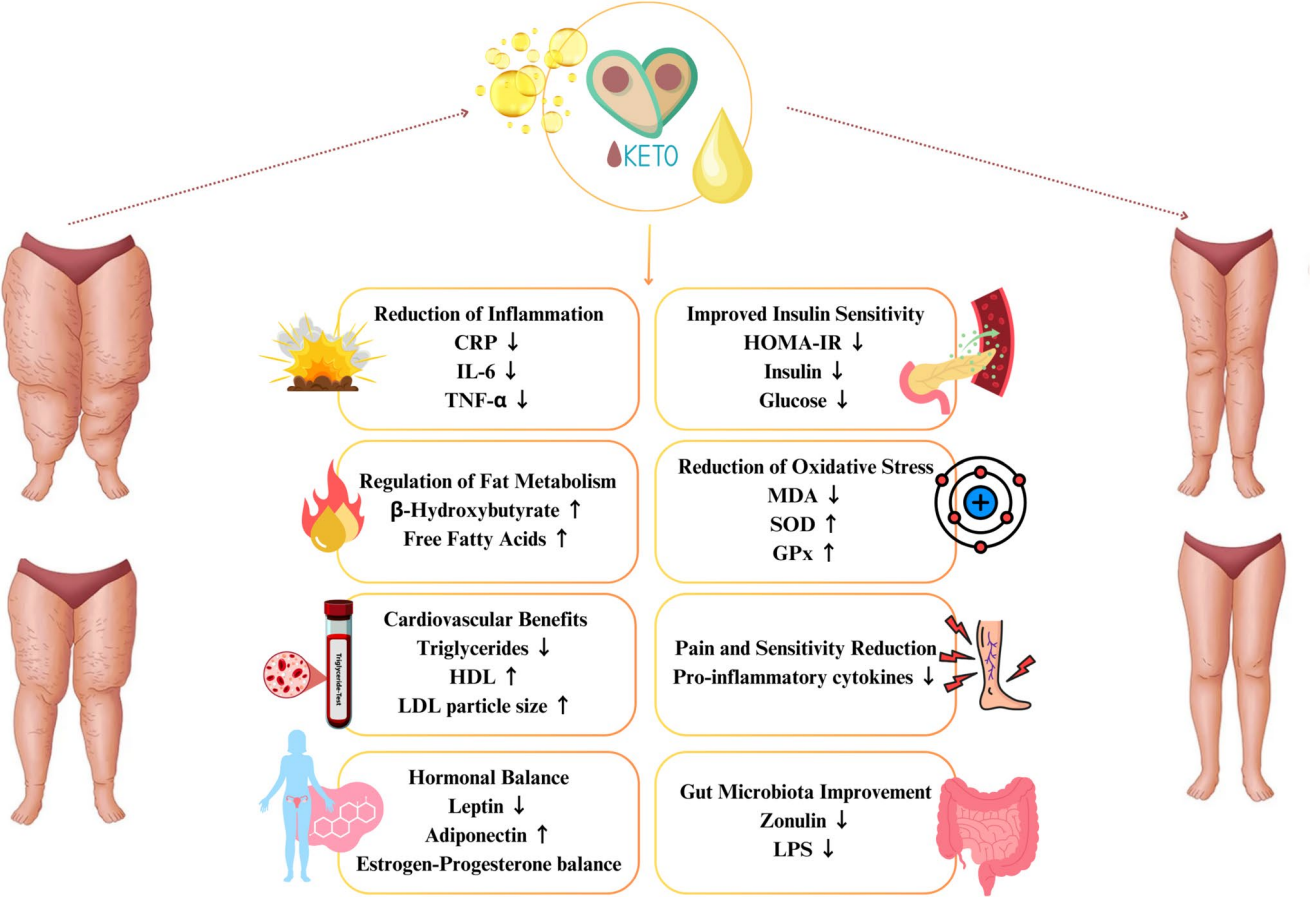
The mechanisms of action of the ketogenic diet on lipedema

Recent studies (2018–2025) (Table 3) consistently report that ketogenic diets, especially when combined with other interventions or nutraceuticals, can reduce body fat, improve pain, and enhance quality of life in patients with lipedema. Both pilot studies and systematic reviews highlight significant improvements in anthropometric and metabolic parameters, though larger and longer-term studies are still needed for confirmation. The mechanisms of action of the ketogenic diet on lipedema are presented in Fig. 2.

**Table 3:** Some studies from 2018–2025 summarizing the effects of ketogenic diets on lipedema patients

Reference	Study Design	Intervention	Duration	Effects
[78]	Women with lipedema (*n* = 9)	High fat, low carbohydrate diet (fat 70–75%, protein 20%, carbohydrate 5–10%)	7 weeks	Significant body weight loss and reduction in pain
[16]	Women with lipedema (*n* = 34)	Modified Mediterranean ketogenic diet	10 weeks	Reduction in weight, BMI and waist circumference Improvement in skin texture on the legs
[89]	A 32-year-old woman diagnosed with lipedema type IV and V, stage II-III	A calorie deficit of 200–250 kcal, carbohydrate intake no more than 25 g per day, ratio between proteins and fats between 1:1 and 1:2, average intake of 1300 kcal divided into 30% from proteins, 66% from fats and 4% from carbohydrates	14 months	Decrease in body circumference measurements, loss of body weight (−41 kg), pain and an improvement in overall quality of life
[88]	Women with lipedema (*n* = 91)	LCHF and MCMF	Single case, 22 months KD	Decrease in body weight and all anthropometric parameters, decrease in pain in extremities, improvement in mobility
[84]	Women with lipedema (*n* = 52)	LCHF with anti-inflammatory properties	28 weeks	Decrease in body weight, body fat mass, pain levels, anthropometric parameters
[80]	Women with lipedema (*n* = 48)	Personalized calorie-restricted, low-carb, high-fat diet	28 weeks	Decrease in body weight, TGs Increase in HDL-C, liver parameters, Improvement in glucose tolerance and fasting insulin levels
[92]	Women with lipedema (*n* = 70)	Low energy, low carbohydrate diet	8 weeks	Body weight loss Reduction in pain
[15]	*n* = 22 patients, 3 groups (MMKD + carboxytherapy, MMKD alone, carboxytherapy alone)	Modified Mediterranean-Ketogenic Diet and Carboxytherapy as Personalized Therapeutic Strategies in Lipedema: A Pilot Study	10 weeks	Ketogenic diet led to weight/fat loss, improved pain, sleep, and skin; best results with combined therapy
[12]	The efficacy of ketogenic diets (low carbohydrate; high fat) as a potential nutritional ıntervention for lipedema: A Systematic review and meta-analysis	Systematic review/meta-analysis, 7 studies, 329 women	mean 15.85 weeks	Significant reductions in BMI, body weight, waist/hip circumferences, and pain sensitivity
[76]	Patients with lipedema (*n* = 100)	Pilot study, women with lipedema	13 weeks	Significant weight loss (~ 4.6 kg), reduced pain during diet phase, improved quality of life; pain returned to baseline after diet ended
[81]	Ketogenic diet as a potential intervention for lipedema	Review/discussion of mechanisms and evidence		KD may alleviate pain, fat deposition, and improve quality of life in lipedema
[93]	The relationship between fat gain and lipedema progression was examined	*n* = 100 patients with lipedema	4.6 years	The more the BMI increased, the more the lipedema progressed WHtR increase
[94]	Influence of ketogenic diet and nutraceutical correction in the complex treatment of lower limbs lipedema	60 patients, 2 groups (LCD vs. modified Atkins KD + nutraceuticals)	4 weeks	KD group had greater fat loss, improved metabolic markers, reduced leptin/insulin, and edema
[13]	The effect of a low-carbohydrate diet on subcutaneous adipose tissue in females with lipedema	Clinical trial	Not specified	Low-carb diet reduced calf subcutaneous fat, circumference, and pain; both diet and control groups lost weight and fat mass
[91]	The effect of a low-carbohydrate diet on subcutaneous adipose tissue in females with lipedema	RCT; 13 women		LCD reduced subcutaneous fat, calf circumference, and pain; both diets reduced weight
[95]	This study compared the biochemical parameters of women with lipedema and women with overweight/obesity	108 women, including 53 with lipedema and 55 with lifestyle-induced overweight/obesity	~ 16 weeks	Women with lipedema exhibited a more favorable metabolic profile than those with overweight/obesity. Dyslipidemia was observed in ~ 50% of the lipedema group, compared to nearly 70% in the overweight/obesity group

Women with lipedema have demonstrated the negative impact of the disease on their mental health and quality of life, underlining the need for more individualized and empathetic therapeutic approaches [82]. It has been proven that weight loss does not affect the prognosis of lipedema caused by fat accumulation. Lipedema resistance to dietary treatment is high, with 95% of patients unable to lose weight in lipedema areas. In individuals with insulin resistance, increased lipolysis and impaired lipogenesis in adipose tissue lead to the release of cytokines and lipid metabolites, ultimately increasing insulin resistance. Therefore, since no specific diet has been developed for lipedema to date, an isoglycemic diet appears appropriate [25]. It has been hypothesized that the ketogenic diet may be effective in the treatment of lipedema in terms of weight loss, reduction of edema, modulation of the inflammasome, and subsequent improvement of the redox status [15]. The diet may partially affect systemic inflammation through its effect on weight. High BMI and obesity are associated with low-grade inflammatory status and elevated levels of circulating inflammatory markers. Therefore, weight loss leads to a reduction in inflammatory cytokines, including CRP, IL-6, and TNF-α. These cytokines and metabolic effects intersect with inflammatory skin disease pathways [83]. Improved benefits of the modified Mediterranean diet compared to the ketogenic diet have been reported, including improved skin texture, reduced pain, and better sleep quality [16]. Another study reported improvements in health-related quality of life and no significant adverse effects in patients with lymphedema following a ketogenic diet [84]. Cifarelli et al. found that in women with obesity and lipedema (Obese-LIP) who lost weight with a moderate (~ 9%) diet, compared to women matched for age, BMI, and total body fat percentage, there was ~ 23% more leg fat mass, ~ 11% less android-gynoid ratio, and ~ 48% more insulin sensitivity. Weight loss improved insulin sensitivity and reduced total fat mass with similar reductions in abdominal and leg fat mass. Moderate dietary weight loss improved metabolism and reduced lower-body adipose tissue mass [85].

Dietary supplements that reduce fat tissue and increase lean body mass may be an effective adjunct in the treatment of lipedema. Reducing fat tissue and increasing lean body mass can potentially improve body image, decrease pain, and enhance mobility in individuals with lipedema. The use of certain dietary supplements may help reduce total body fat and increase lean body mass. This may prevent the invasive application of microcannulated tumescent lipedema reduction surgery, including suction lipectomy, which is currently the most effective treatment for lipedema [21]. Estrogens are thought to play an essential role in the progression of lipedema. Aromatase, an enzyme produced by adipocytes, converts androgens into estrogens. Therefore, dietary supplements that alter hormonal levels may be an effective strategy for the treatment of lipedema. Supplementation of molecules acting on adrenergic receptors may help increase metabolic rate and fat burning and accelerate weight loss in women with lipedema. Adrenergic enhancers include 7-Keto-DHEA and yohimbine. 7-Keto-DHEA causes long-term changes in body levels of epitestosterone, testosterone, estradiol, and other steroid hormones and is reported to stimulate thermogenesis, converting stored fat into ATP and directing heat production. Indeed, these supplements are known to increase energy expenditure by enhancing fat mass loss, increasing fat metabolism, and impairing fat absorption [86]. Current evidence suggests that general weight loss does not effectively reduce lipedema fat, and in some cases, it may exacerbate the disproportion between the upper and lower body. This is because lipedema is characterized by a specific pattern of fat distribution that is resistant to conventional weight loss methods. The use of fat-burning supplements, often marketed as a solution for weight loss, may not only be ineffective for lipedema but could also pose potential health risks. Lipedema is a condition marked by an abnormal accumulation of fat in the lower body, which is resistant to diet and exercise. Weight loss can improve metabolic health and reduce total fat mass, it does not significantly alter the disproportionate fat distribution seen in lipedema [85].

Fat-burning supplements, which often contain ingredients like caffeine, green tea, and carnitine, are marketed to enhance weight loss by boosting metabolism and promoting fat breakdown. However, their effectiveness in treating lipedema is questionable. A study found that the use of dietary supplements containing a mixture of plant components for weight loss had a negative effect on glucose metabolism and insulin resistance, suggesting potential health risks associated with their use.The effects of fat burners in human studies are sometimes contradictory, and further research is needed to assess their safety and efficacy in lipedema management [86]. Specific dietary interventions, such as ketogenic and modified Mediterranean diets, have shown promise in managing lipedema by reducing inflammation and improving metabolic health. These diets, combined with regular physical exercise and stress management, may offer a more effective approach to managing lipedema symptoms [13]. Personalized treatment strategies that consider the unique characteristics of lipedema, such as its resistance to conventional weight loss and the presence of inflammation, are crucial for effective management. While general weight loss and fat-burning supplements may not be effective for reducing lipedema fat, it is important to consider alternative approaches that address the specific needs of individuals with lipedema. Personalized dietary and lifestyle interventions, along with further research into the condition's pathophysiology, may provide more effective solutions for managing lipedema.

The Mediterranean diet is emphasized in the guideline due to its well-documented health benefits, including anti-inflammatory properties and cardiovascular health improvements. This diet is rich in fruits, vegetables, whole grains, and healthy fats, particularly olive oil, which may help manage lipedema symptoms by reducing inflammation and promoting overall health. The strong consensus for the Mediterranean diet reflects its broad acceptance and evidence supporting its role in managing chronic conditions, including lipedema [10].

Long-term consumption of KD can alter mitochondrial function mainly by reducing ROS production, increasing UCP expression, and promoting ATP production to improve mitochondrial activity [87]. The VLCKD is divided into several phases. The first phase of the VLCKD, also known as the induction phase, is characterized by a very low-calorie diet (650–700 kcal/day) with a low carbohydrate content (< 30 g/day from vegetables) and fat (only 20 g/day, plus olive oil). VLCKD may aid in weight loss and improve inflammation [15].

### Clinical Evidence

Sørlie et al. found that a low-carbohydrate, high-fat caloric diet contributed to significant weight loss and a significant reduction in waist, hip, and thigh circumference in individuals with lipedema over 13 weeks [78]. In another study, it was observed that the LCHF (low carbohydrate, high fat) diet contributed to the reduction of body weight, body fat, waist circumference, hip circumference, and thigh and calf circumferences compared to the MFMC (medium fat, medium carbohydrate) diet in women, decreased pain in the extremities, and improved mobility in the LCHF group [88]. Overweight or obese women with lipedema who were given an LCHF diet with anti-inflammatory properties to adhere to for a period of 7 months experienced a body weight reduction of 12.9%, with the greatest reduction found in body fat mass. Improvements were observed in several parameters except ankle circumferences, and pain levels were significantly reduced following the LCHF diet [84]. In another study, adult female patients with lipedema and obesity were given LCD for 8 weeks, and the LCD group was found to have greater body weight loss and reduction in pain compared to the control group [14]. Cannataro et al. ensured that a 32-year-old female patient with lipedema types IV and V, stage II-III, followed a diet of approximately 1,300 kcal per day for six months. This diet was planned to consist of 66% fat, 30% protein, and 4% carbohydrates. The patient lost approximately 20% of her body fat mass, resulting in a weight loss of 41 kg.

In addition, body circumferences showed a decrease in all regions, and biochemical-clinical parameters showed that kidney and liver function were not adversely affected [89]. Another study reported a possible association between the presence of HLA-DQ2 or HLA-DQ8 or both and inflammation seen in lipedema, especially when combined with gluten intake. Lipedema patients seeking medical attention have a prevalence of HLA-DQ2 and HLA-DQ8. Given the role of gluten in inflammation, further research is needed to determine whether this association supports the benefit of removing gluten from the diet in the management of lipedema symptoms [90]. Another study demonstrated a significant reduction in anthropometric measurements and body composition measurements and a significant association with low-carbohydrate, high-fat ketogenic diets in the management of lipedema [13]. A high-CHO diet containing more than 45% of total daily energy has been found to contribute to water retention, which contributes to excessive lymphatic load. In addition, KD in lipedema may increase lymphatic transport due to decreased lymphatic endothelial cell permeability. Therefore, KD may reduce fluid load due to decreased attack on capillary endothelial lymph. The hypothesis has been proposed that carbohydrate restriction, when encouraged as part of a well-formulated KD, may reduce excessive tissue water content in lipedema when combined with fat consumption [15]. The ketogenic diet, characterized by low carbohydrate and high fat intake, has shown promise in managing lipedema by reducing body fat and inflammation [15]. Weight loss and symptom improvement in lipedema patients following a ketogenic diet, with some reports of substantial reductions in pain and improvements in quality of life [78, 89]. The guideline's consensus on the ketogenic diet acknowledges its potential benefits but also highlights the need for further research to confirm long-term safety and efficacy [15, 16].

In the long term, individual responses to ketogenic diets may vary, and factors such as genetic predisposition, baseline metabolic health, and dietary adherence may influence outcomes, and while it has been reported to improve lymphatic function in some patients with lymphedema, the reduction in edema volume was not the same in all participants [91]. Although the ketogenic diet, particularly its high-fat, low-carbohydrate variants, is a promising nutritional intervention and approach for the treatment of lipedema, it is important to note that this condition is primarily characterized by painful fat accumulation in women, that individual responses may vary, and that further studies are needed to establish long-term efficacy and safety. In addition, it may suggest that a more balanced approach that includes a variety of dietary strategies may also yield beneficial results for patients with lipedema.

Lipedema is a chronic and debilitating disease affecting mostly women, and its diagnosis and etiology remain unclear. Often misdiagnosed as obesity or lymphedema, lipedema is resistant to lifestyle interventions. Their effects in human studies are sometimes contradictory, and further studies should test their efficacy in the treatment of lipedema. Long-term implementation of a ketogenic lipedema diet or low-carbohydrate lipedema diet can be challenging. It is very difficult to establish the safety and long-term effectiveness of a very low-calorie ketogenic diet. However, it has been suggested that a very low-calorie ketogenic diet may be more effective than other dietary changes in the treatment of lipedema.

### Comparative Efficacy and Considerations

There is no clear consensus on the most effective dietary approach for lipedema treatment. The most recent German S2 guideline (2024) provides a strong consensus recommendation for the Mediterranean diet in lipedema management and a consensus for the ketogenic diet. This reflects a growing recognition of the role of dietary interventions in managing lipedema, a chronic condition characterized by abnormal fat accumulation, primarily in the lower body. The guideline aims to offer a comprehensive approach to treatment, integrating various professional perspectives to optimize patient outcomes. The Mediterranean diet is recommended for its overall health benefits, while the ketogenic diet is noted for its potential in reducing inflammation and improving symptoms in lipedema patients.

While the Mediterranean diet is recommended for its general health benefits, the ketogenic diet is noted for its specific impact on lipedema symptoms, particularly in reducing inflammation and adipose tissue [13]. Some studies suggest that the ketogenic diet may be more effective than the Mediterranean diet in certain aspects of lipedema management, such as reducing pain and improving body composition [15, 16]. However, the ketogenic diet's long-term effects and safety remain areas of active research, and its implementation should be carefully monitored [91].

In considering these dietary approaches, it is important to recognize the individual variability in response to dietary interventions. While the Mediterranean diet is broadly recommended for its health benefits, the ketogenic diet may offer specific advantages for some lipedema patients, particularly in managing symptoms and reducing inflammation. However, the need for personalized treatment plans and further research into the long-term effects of these diets is crucial to optimize lipedema management strategies. A significant limitation is that current behavioral interventions for lipedema are generally ineffective. There is also insufficient information in the literature regarding dietary patterns and modifications in lipedema.

## Limitations and Future Directions

Research on lipedema has several limitations that impact the robustness and applicability of the findings. Most studies focus on specific groups or regions. This limits the generalizability of results to broader demographics. Many studies have been conducted with relatively small sample sizes, which can reduce the reliability and applicability of findings to larger populations. There is a need for longer-term studies to evaluate the effects of lifestyle changes, as current research often overlooks this aspect. The clinical importance of lipedema levels in specific metabolic diseases and obesity and their utility as biomarkers for diagnostic or prognostic purposes have not been fully validated. Further confirmation studies are necessary to establish their roles in clinical practice. Limited studies suggest that the association of lipedema may also be involved in inflammatory processes, hormonal changes, or genetic predispositions. In adition, the fact that the lipedema is still unknown and the mechanisms of action are not clear and concise, and that publications on food, nutrition, and diet models are insufficient, restricts the interpretation of some results. Future research should focus on these factors to clarify their roles. To better understand the significance of lipedema, more comprehensive in vivo*, *in vitro, and human studies are warranted.

Lipedema is a progressive disease characterized by abnormalities in the distribution of adipose tissue and chronic inflammation, usually seen in women, and negatively affects the quality of life of individuals with symptoms such as pain, swelling, and limitation of movement. Current treatment approaches include surgical interventions, compression therapies, and physical rehabilitation methods, but nutrition-based strategies are increasingly being investigated. In this context, KD attracts attention due to its high fat, sufficient protein, and low carbohydrate content, reducing insulin resistance by triggering a state of ketosis in energy metabolism, suppressing systemic inflammation, and accelerating lipolysis processes. It is thought that the ketogenic diet, which has the potential to alleviate edema, inflammation, and pain in lipedema, may improve physical function and quality of life by improving symptom management of individuals. To increase the number of clinical studies in the literature, this study will contribute to raising awareness and better understanding of the relationship between health and ketogenic diet. More randomized controlled studies on the efficacy and safety of this approach are needed, and it is anticipated that the ketogenic diet will find a wider application area in the treatment of lipedema within a personalized treatment paradigm in the future.

## Conclusions and Recommendations

Lipedema is a chronic and progressive disease whose pathogenesis is not fully understood but is thought to be related to the interaction of genetic predisposition, hormonal factors, and inflammatory processes. The symptoms of the disease cover a wide range of conditions, including painful fat accumulation in the lower extremities, edema, and functional limitation, requiring multidisciplinary management. Existing literature suggests that dietary and lifestyle interventions have an important place in the management of lipedema. In this context, the ketogenic diet is an approach that attracts attention due to its metabolic effects. The potential effects of the ketogenic diet on lipedema have been attributed to various biological mechanisms. The diet's effects of increasing insulin sensitivity and decreasing inflammation overlap with the pathophysiology of lipedema. Moreover, the fact that the ketogenic diet increases fat oxidation by altering energy metabolism may be an effective strategy to reduce body fat mass.

Advanced techniques like metabolomics and lipidomics have been employed to identify potential biomarkers and metabolic disturbances specific to lipedema, offering promising diagnostic tools. Recognizing lipedema as distinct from obesity is crucial for appropriate management and treatment. This distinction is important as lipedema patients may not benefit from traditional obesity treatments and may require specialized interventions. Understanding the unique metabolic profile of lipedema can guide more effective treatment plans. While the metabolic profile of lipedema appears distinct from obesity, the condition's pathogenesis remains poorly understood. Hormonal factors are believed to play a significant role, given its prevalence among women. Additionally, the relationship between lipedema and lymphatic function is still debated, with some suggesting that lipedema may predispose individuals to lymphedema due to increased pressure on lymphatic vessels [31, 32]. Further research is needed to elucidate these mechanisms and improve diagnostic and treatment strategies.

However, there is limited data in the literature on the long-term applicability and safety of this dietary model. Given the heterogeneous nature of lipedema, further clinical trials are needed for individual adaptation of the ketogenic diet and evaluation of its efficacy. Personalized treatment strategies that consider the unique characteristics of lipedema, such as its resistance to conventional weight loss and the presence of inflammation, are crucial for effective management. While general weight loss and fat-burning supplements may not be effective for reducing lipedema fat, it is important to consider alternative approaches that address the specific needs of individuals with lipedema.

In conclusion, the ketogenic diet is a promising dietary approach for the treatment of lipedema. However, more controlled studies are needed to demonstrate the effects of this diet on the symptoms and progression of the disease. In the implementation process, factors such as metabolic status, stage of the disease, and lifestyle should be taken into account, and a personalized treatment plan should be created under the guidance of a health professional. In addition to diet, multidisciplinary approaches such as regular exercise, compression therapy, and surgical interventions when necessary remain important in the management of lipedema.

## Sample Case

### General İnformation

**Table:** 

Patient's name/surname	M.A		
Gender of the patient	Female		
Patient age (years)	34		
Occupation status	Secretary		
Marital status	Single		
Diagnosis	Stage 2 Lipedema		
Symptoms	Edema and thickening of the lower extremities		
Body weight (kg)	107	BMI (kg/m^(2^))	39.3
Height length (cm)	165	Ideal BMI (kg/m^(2^))	22

### Medical Anamnesis

**Table:** 

Resume	The patient complained of disproportionate body weight gain since adolescence and swelling especially in the leg region. She states that she has difficulty losing weight with low-calorie diets, but edema increases periodically
Family history	There was a history of obesity in the father, diabetes in the mother and lipedema in the aunt
Medicines used	The patient uses anti-inflammatory and circulatory supplements under the supervision of a doctor
Smoking-alcohol use	He does not smoke or drink alcohol
Surgical history (if available)	Since venous insufficiency is common in individuals with lipedema, removal of varicose veins has undergone surgery

### Nutritional History and Assessment of Food Consumption Status

The patient stated that she had previously followed low-calorie diets, but there was no significant thinning, especially in the lower body. She stated that he observed that his complaints of edema increased after carbohydrate consumption and that he experienced sugar crises from time to time. Water consumption is insufficient and averages 1.5 L/day.

### Clinical Findings, Physical Examination

The patient presented with complaints of painful fat accumulation, edema and sensitivity to touch, especially in the lower extremities. She has limited mobility in activities of daily living and has difficulty losing weight. There are signs of impaired lymphatic circulation and inflammation.

### Calculation of Daily Energy and Nutrient Requirements of the Patient

**Table:** 

BMR (kcal)	Practical Formula: 72 (kg) × 0.95 kcal/kg × 24 = 1641 kkal
Total energy requirement (kcal)	TEH = BMH X F.A = 1641 × 1.3 = 2134 kkal It was considered appropriate to start with an average of 1700 kcal

### Recommended Diet, Menu Planning and Menu Energy and Nutrient Calculation and Recommendations

**Table:** 

Energy-nutrients		Amount	Percentage of energy from carbohydrate, protein and fat (%)
Energy (kkal/day)		1700	
Protein (g/day)		30	7
Carbohydrate (g/day)		18	4.2
Fat (g/day)		170	88.9
**Lipedema Diet at a 3:1 KD Ratio of 1700 kkal**			
**Breakfast**	1 cup of light tea (unsweetened) Eggs with cream (2 eggs + 2 tablespoons cream) 24 olives 1 tablespoon olive oil		
**Birdhood**	10 medium hazelnuts		
**Noon**	Creamy vegetable dish **(6 tablespoons of vegetables prepared with 7 teaspoons of cream)** Creamy avocado appetizer **(1/2 avocado + 4 teaspoons cream)** 1 tablespoon olive oil **(as an extra source of fat)**		
**Afternoon**	3 whole walnuts		
**Evening**	Creamy chicken sauté **(40 g chicken + 8 teaspoons cream)** 1 tablespoon olive oil **(as an extra source of fat)**		
**Night**	10 medium hazelnuts		

### What are the Characteristics of the Ketogenic Diet?

Support fat burning,be able to improve insulin levels, reduce pain, bloating and edema, hunger control, your metabolism should up, body composition should improve, ınclude foods with low glycemic load,.less carbohydrates, less fat should be at a level to store, improve blood circulation, support psychological support and energy levels, reduce edema and inflammation, ımprove lipid profile, ıt should be sustainable.

### Patient Appropriate Nutrition Recommendations

Meals should be taken regularly and quantities should be observed. Consumption of healthy fats such as avocado, olive oil, walnuts, almonds is important and should be included frequently in the diet.Good protein sources such as eggs, red meat and chicken should be preferred. Fresh and seasonal vegetables should be preferred. Seasonal fish should be consumed once a week in terms of omega-3 content.

### Foods to Avoid

Foods with high sugar content (sweets, cakes, confectionery, ready-made fruit juices containing refined sugar, sugary snacks (candies, energy bars, fruit rolls, ice cream, sorbet, etc.), sugary sweeteners (agave syrup, high fructose corn syrup, honey and artificial sweeteners), sweet condiments (jams, jellies, preserves, sauces, syrups, etc.), sweet drinks, sugar-sweetened and diet drinks. foods high in salt (packaged chips, fast food, pickles, soy sauce, etc.), processed and packaged foods (frozen foods, instant soups, processed meat products), alcohol, beer, liquor, mixed drinks, wine, etc., saturated and trans fats, margarine, fried foods, packaged pastries, soy-based processed foods, highly processed soy products, foods of unknown composition.

## Key References

- Lomeli LD, Makin V, Bartholomew JR, Burguera B. Lymphedema vs lipedema: Similar but different. Cleve Clin J Med. 2024 Jul 1;91(7):425–436. 10.3949/ccjm.91a.23084.
  - Lymphedema and lipedema are chronic, debilitating disorders that most commonly affect the upper and lower extremities. Although they can appear similar, they differ in important ways, which the authors of this article review and contrast. This study explains the confusion between lymphedema and lipedema.
- Lundanes J, Gårseth M, Taylor S, Crescenzi R, Pridmore M, Wagnild R, et al. The effect of a low-carbohydrate diet on subcutaneous adipose tissue in females with lipedema. Front Nutr. 2024 Nov 7;11:1484612. 10.3389/fnut.2024.1484612.
  - A LCD has the potential to reduce SAT and pain in females with lipedema, despite a reduction in muscle mass in lipedema affected areas in both diet groups. Further studies are needed to confirm these findings and explore potential mechanisms. This study provides a different perspective on our research regarding its subject matter.
- Cifarelli V, Smith GI, Gonzalez-Nieves S, Samovski D, Palacios HH, et al. Adipose Tissue Biology and Effect of Weight Loss in Women With Lipedema. Diabetes. 2025 Mar 1;74(3):308–319. 10.2337/db24-0890.
  - Women with obesity and lipedema have decreased expression of genes related to lymphatic/vascular function and increased expression of genes related to fibrosis and inflammation in thigh compared with abdominal subcutaneous adipose tissue; weight loss increased insulin sensitivity and decreased leg fat but did not affect adipose tissue inflammation or fibrosis. Weight loss should be the first-line therapy for women with obesity and lipedema. These results inspired our study.

## Data Availability

No datasets were generated or analysed during the current study.

## Abbreviations

AcAc: Acetoacetate
AKR1 C1: Aldo-keto reductase family 1 member C1
ATP: Adenosine triphosphate
BMI: Body mass index
CAV1: Caveolin 1
Ckd: Classical ketogenic diet
CRP: C-reactive protein
CVD: Cardiovascular diseases
DM: Diabetes mellitus
ER: Estrogen
GH: Growth hormone
HMG-CoA: 3-Hydroxy-3-methylglutaryl-CoA
IL-6: İNterleukin-6
KD: Ketogenic diet
LCHF: Low carb, high fat
LGIT: Low glycemic index therapy
MAD: Modified Atkins diet
MCTKD: Medium-chain triglyceride ketogenic diet
MIF: Macrophage migration inhibitory factor
miRNA: MicroRNA
ROS: Reactive oxygen species
SAT: Subcutaneous adipose tissue
SVF: Stromal vascular fraction
TG: Triglyceride
TNF-α: Tumor necrosis factor-alpha
UCP: Uncoupling proteins
VLCKD: Very low-calorie ketogenic diet
βHB: β-Hydroxybutyric acid
